# Comparing the Potential of Near- and Mid-Infrared Spectroscopy in Determining the Freshness of Strawberry Powder from Freshly Available and Stored Strawberry

**DOI:** 10.1155/2019/2360631

**Published:** 2019-03-17

**Authors:** Da Wang, Wenwen Wei, Yanhua Lai, Xiangzheng Yang, Shaojia Li, Lianwen Jia, Di Wu

**Affiliations:** ^1^College of Energy and Power Engineering, Shandong University, Jinan 250061, China; ^2^Jinan Fruit Research Institute, All China Federation of Supply and Marketing Cooperatives, Jinan 250014, China; ^3^College of Agriculture & Biotechnology/Zhejiang Provincial Key Laboratory of Horticultural Plant Integrative Biology/The State Agriculture Ministry Laboratory of Horticultural Plant Growth, Development and Quality Improvement, Zhejiang University, Zijingang Campus, Hangzhou 310058, China

## Abstract

The quality of strawberry powder depends on the freshness of the fruit that produces the powder. Therefore, identifying whether the strawberry powder is made from freshly available, short-term stored, or long-term stored strawberries is important to provide consumers with quality-assured strawberry powder. Nevertheless, such identification is difficult by naked eyes, as the powder colours are very close. In this work, based on the measurement of near-infrared (NIR) spectroscopy and mid-infrared (MIR) spectra of strawberry powered, good classification results of 100.00% correct rates to distinguish whether the strawberry powder was made from freshly available or stored fruit was obtained. Furthermore, partial least squares regression and least squares support vector machines (LS-SVM) models were established based on NIR, MIR, and combination of NIR and MIR data with full variables or optimal variables of strawberry powder to predict the storage days of strawberries that produced the powder. Optimal variables were selected by successive projections algorithm (SPA), uninformation variable elimination, and competitive adaptive reweighted sampling, respectively. The best model was determined as the SPA-LS-SVM model based on MIR spectra, which had the residual prediction deviation (RPD) value of 11.198 and the absolute difference between root-mean-square error of calibration and prediction (AB_RMSE) value of 0.505. The results of this work confirmed the feasibility of using NIR and MIR spectroscopic techniques for rapid identification of strawberry powder made from freshly available and stored strawberry.

## 1. Introduction

Strawberries are known to be a natural source of phenols and other bioactive substances in the world of phytonutrients. Strawberry powder obtained by freeze-drying retains most nutrients of fresh strawberries and is an excellent source of dietary fiber, antioxidants, and vitamin C. As a natural and safe pigment and flavoring agent, strawberry powder can be used to introduce a rich nutrient and natural strawberry flavor boost to the diet in a natural way, such as candies, jelly, custard, yoghurt, milkshakes, sorbets, and smoothies. Strawberry powder as diet supplements shows health benefits. In a trial with healthy human subjects, a 3-week dietary intervention with strawberry powder reduced plasma concentrations of cholesterol and small HDL-cholesterol particles and increased LDL particle size in obese subjects [1]. In the high-fat diet-induced obese C57BL/6 mice, strawberry powder decreased the blood glucose level and lowered the plasma C-reactive protein [2]. These results indicated a potential role of strawberry powder on reducing the risks associated with obesity and diabetes. In another work, the anthyocyanins in the strawberry powder was considered as the functional components in reducing obesity [3].

The good flavor, health benefits, and high economic value of strawberry powder depend on the freshness of strawberries. High-quality strawberry powder needs to be made from fresh strawberries. The quality of the stored strawberries is not as good as the fresh strawberries. Therefore, the strawberry powder made from the stored strawberries has worse quality than those made from the freshly available strawberries. However, because the price of stored strawberries is lower than fresh strawberries, some producers deliberately made strawberry powder from stored strawberries and told the consumers that the powder is made from fresh strawberries for economic benefit. This kind of behavior seriously violates the rights and interests of consumers. Nevertheless, for consumers, it is difficult to distinguish whether the strawberry powder is made from freshly available or stored strawberries by the naked eyes, as the powder colours are very close. The identification by chromatography techniques is time-consuming and labor-intensive. Therefore, there is a need for a technique capable of quickly identifying whether the strawberry powder is made from the fleshly available or the stored strawberries. Moreover, considering that the quality deterioration of strawberry stored in a short-term period is not as much serious as those stored in a long-term period, the strawberry stored in a short-term period can also be used to produce strawberry powder that does not have high quality requirements, as this kind of powder's quality is not very bad and can have low price, and will be welcomed by some consumers with low income. Therefore, it is more meaningful to predict the storage days of the strawberries that the strawberry powder is made from.

Near-infrared (NIR) spectroscopy has become one of the fastest growing and most compelling modern quantitative analysis technologies for the quality inspection of agricultural products and food [4–6] due to its short analysis time, no sample pretreatment, nondestructive and nonpolluting nature, and low cost. NIR has been used for powder adulteration and quality prediction [7–9], but there was no adulteration research on strawberry powder. On the contrary, mid-infrared (MIR) spectroscopy is the analysis of mid-infrared light interacting with functional groups in molecules [10, 11]. MIR spectra provide richer frequency and intensity information than NIR because the spectral absorption band in the NIR region is only the overtone and overtone combination of stretch vibration and bent vibration of higher energy bonds, such as C–H, O–H, and N–H in the organic matter in the MIR region [12]. Although traditional MIR spectral measurement requires the use of a specially purified salt (usually potassium bromide) to finely grind a certain amount of sample, which makes the MIR measurement destructive and time-consuming, when the MIR spectrometer is equipped with the attenuated total reflectance mode, it allows samples to be tested directly in solid or liquid form without further preparation, which makes the MIR measurement also rapid and almost nondestructive. When the NIR or MIR spectral model has been established, it is only necessary to measure the NIR or MIR spectrum of the new sample and introduce it as an input into the established spectral model, which can be used to quickly predict the quality of the new sample [5, 13].

The object of this study was to compare the potential of using NIR and MIR techniques to identify strawberry powder made from freshly available and stored fruit in a rapid way. After acquiring RGB images of strawberry powder, the NIR and MIR spectra of strawberry powered were used to distinguish whether the strawberry powder was made from the freshly available or the stored fruit. Furthermore, the NIR and MIR spectra were used to predict the storage days of strawberries that produce strawberry powder. Two calibration methods and four variable selection strategies were used for the spectral analysis and their performances were compared. It should be noticed that although there are many works using NIR to determine firmness, water content, soluble solids content, pH, and colour of various fruits and vegetables [14–16], these works mainly focused on the study of fresh fruit and vegetables rather than fruit powder. In other words, in these works, the determination of the quality parameters of fruit or vegetables was based on the spectra of fruit or vegetable itself. On the contrary, this work determined whether the fruit was freshly available or stored based on the spectra of fruit powder. To the best of our knowledge, neither NIR nor MIR spectroscopic techniques have been used for rapid identification of strawberry powder made from freshly available and stored fruits.

## 2. Materials and Methods

### 2.1. Strawberry Powder Preparation

Strawberries (Fragaria x ananassa L. cv. Zhangji) were hand-harvested at commercial maturity from an orchard in Jinan, Shandong Province, China, and transported within 1 h to the laboratory at Jinan Fruit Research Institute. Uniform fruit, free from blemishes, were selected by size, colour, ripeness, and absence of visually apparent infections. All fruits were placed in opened polyethylene boxes at 0 ± 1°C for storage. The storage period lasted for 27 days. Sampling was performed on days 0, 4, 15, 20, and 27 of the storage period. The fruits on the 0^th^ day of the storage period were the fresh fruits. The sampled strawberries were used to make strawberry powder using an LYO-01 vacuum-freeze dryer (Tofflon Freezing Drying Systems, Shanghai, China).

### 2.2. Acquisition of NIR and MIR Spectra of Strawberry Powder

Fourier transform infrared spectrometer equipment (Bruker Vertex 70, Bruker Optics GmbH, Faellanden, Switzerland) with a DTGS detector, KBr beam splitter, and 100 micrometer diaphragm was used to collect NIR (12000–4000 cm^−1^) and MIR spectra (4000–600 cm^−1^) of strawberry powder. Especially for the MIR spectral measurement, a Tensor 27 spectrophotometer (Bruker, Inc., Germany) equipped with a golden gate diamond ATR sampling accessory was used. The NIR and MIR spectrum collection process was performed at room temperature. The spectrum of each sample was collected 16 times using OMINIC software (Version 5.2, Bruker, Inc.) to reduce noise. Each scan took about 5 seconds. The acquired 16 spectra were averaged as the spectrum of the sample. The obtained spectral data had a resolution of 4 cm^−1^ and a signal-to-noise ratio of more than 55000 : 1 (peak-to-peak value). It took 30 min to preheat the spectrometer before spectral acquisition.

### 2.3. Multivariate Analysis

Two multivariate regression methods, namely, partial least squares regression (PLSR) and least squares support vector machines (LS-SVM) were used to establish the quantitative model between the spectral matrix of strawberry powder (*X*) and the storage days of strawberries (*Y*). PLSR is a classical linear regression method, particularly suitable when the number of variables is more than the number of samples [17, 18]. The main principle of PLSR is to project spectral data onto a set of factors called latent variables (LVs) to obtain the most representative spectral features. The number of optimal LVs is determined by the prediction residual error sum of squares calculated by leave-one-out cross validation. At last, the quantitative relationship is established between *Y* and the optimal LVs. LS-SVM is more suitable for dealing with nonlinear regression modeling [19, 20]. LS-SVM is an optimized version of SVM. It solves the problem by converting the convex quadratic programming problem into a set of linear equations. This study used the Radial Basis Function (RBF) as the LS-SVM kernel because its performance is generally superior to other kernels. The optimal values of the LS-SVM parameter *γ* and the RBF kernel function parameter *σ*^2^ were determined by the grid search method with leave-one-out cross validation.

### 2.4. Variable Selection

The collected NIR and MIR data had 2074 and 1762 variables, respectively. Most of these variables were redundant and collinear. To solve this problem, three variable selection algorithms were used to select the optimal variables. They were successive projections algorithm (SPA), uninformative variable elimination (UVE), and competitive adaptive reweighted sampling (CARS). In addition, SPA and UVE algorithms are also often used in conjunction, which is called UVE-SPA, to select optimal variables. The selection strategies of these variable selection algorithms are different. The SPA solves the collinearity problem of spectral data by selecting the optimal variables with the minimal redundancy, thereby greatly reducing the dimensionality of the spectral data [21, 22]. UVE removes uninformative variables by calculating the stability of the PLSR regression coefficients [23]. The variables selected by UVE generally still have dozens or even hundreds. Therefore, it is recommended to further perform SPA calculation based on the variables after UVE selection, so as to obtain variables with rich information and low collinearity. The CARS-based variable selection is mainly based on Darwin's Evolution Theory [24]. CARS first takes the variables with the larger absolute values of the regression coefficients in the PLSR model as the candidate variables, and then determines the optimal variable according to the survival theory of the fittest. In this study, a total of four variable selection methods, SPA, UVE, UVE-SPA, and CARS, were used to select the best variables from the spectral data.

### 2.5. Model Evaluation

When the spectral model is established, its detection accuracy and robustness need to be evaluated. The following indicators were used for evaluating the accuracy of infrared spectroscopy models, including correlation coefficients of calibration and prediction (*r*_C_ and *r*_P_), determination coefficients of calibration and prediction (*R*_C_^2^ and *R*_P_^2^), root-mean-square error of calibration (RMSEC) and prediction (RMSEP), and residual prediction deviation (RPD). The absolute difference between RMSEC and RMSEP (AB_RMSE) was used to evaluate the robustness. A good model should have high precision and strong robustness, that is, high *r*_C_, *r*_P_, *R*_C_^2^, *R*_P_^2^, and RPD values and low RMSEC, RMSEP, and AB_RMSE values are required. The model establishment and variable selection in this study were carried out in software Matlab R2015b (the MathWorks, Natick, USA).

## 3. Results

### 3.1. RGB Imaging of Strawberry Powder

**Figure 1:**
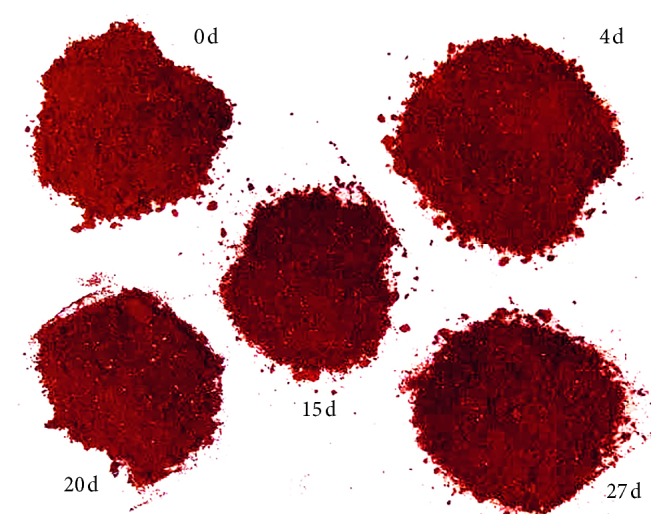
RGB images of strawberry powder made from strawberries with different storage days.

The RGB images of strawberry powder were analyzed before spectral analysis. The measurement of the RGB images was carried out for the strawberry powder made from strawberries with different storage days, as shown in Figure 1. It can be seen that strawberry powder made from strawberries with different storage days had similar appearance and colour. This shows that it was difficult to judge the storage days of the strawberries used to make the strawberry powder by visual observation.

### 3.2. Identification of Strawberry Powder Made from Freshly Available and Stored Fruit

Partial least squares discriminant analysis (PLS-DA) and LS-SVM were used to establish classification models. The reference values of the dependent variable were set −1 and 1 for freshly available and stored fruit, respectively. The results show that when NIR data was considered, the PLS-DA model had the correct rates of 95.95% and 94.59% for calibration set and prediction set, respectively. When MIR data were considered, the PLS-DA model had the correct rate of 100.00% for both calibration set and prediction set. On the contrary, when LS-SVM was used for the model calibration, both NIR and MIR data had the correct rate of 100.00% for both calibration set and prediction set. The above results show that both NIR and MIR could identify strawberry powder made from freshly available and stored fruit.

### 3.3. Prediction of Storage Days Based on NIR Spectra

#### 3.3.1. Calibration with Full NIR Variables

The full-variable NIR spectral detection models for predicting the storage days of strawberry were established by using PLSR and LS-SVM algorithms, respectively. A total of 111 samples from five storage days were used for the data analysis. Two-thirds of the samples constituted the modeling set, and the remaining one-third of the samples constituted the independent prediction set. When the full-variable data of NIR spectra were considered, its PLSR and LS-SVM models obtained good prediction results (Table 1). *R*_P_^2^ values of both models were higher than 0.9, and RMSEP values of both models were lower than 3.0. In addition, when comparing the calculation results of LS-SVM and PLSR model in the calibration set, it was found that the value of *R*_C_^2^ and RMSEC in the LS-SVM model were 1 and 0.001, respectively. The results of the calibration set of the LS-SVM model were much better than the results of the prediction set, indicating that the LS-SVM model had a certain degree of overfitting. On the contrary, the results of the calibration set of the PLSR model were similar to those of the prediction set, indicating that the PLSR model had better robustness.

#### 3.3.2. Calibration with Selected NIR Variables

When quantitative models were established based on the NIR spectral data of full variables, a large number of unneeded and redundant variables were also input into the models. These useless variables contributed little to the model establishment and caused large calculation burden and time in the model establishment process and may even reduce the prediction accuracy of the models. Therefore, selecting a small number of important variables from the full variables was important in reducing the spectral dimension and improving the accuracy and robustness of the models. Four variable selection strategies of SPA, UVE, UVE-SPA, and CARS were carried out to select the optimal variables for predicting the storage days. The selected optimal variables were considered as the input variables to establish the quantitative PLSR and LS-SVM models. The prediction results of PLSR and LS-SVM models based on the optimal variables are shown in Table 1. There were 2074 variables in the NIR spectra. However, only 3 variables were selected after the SPA variable selection. The number of variables was only 0.14% of the total number of full variables. The quantitative model of PLSR and LS-SVM based on the optimal variables selected by SPA was established to predict the storage days of strawberry powder. The accuracy of PLSR and LS-SVM models established based on optimal variables was similar to the corresponding full-variable models. On the contrary, there was no overfitting in the SPA-LS-SVM model, showing that the SPA selection improved the LS-SVM model's robustness. By comparing the results of the SPA-PLSR model and SPA-LS-SVM models, the latter had a better prediction with *R*_P_^2^ of 0.940 and RMSEP of 2.359. When another algorithm of UVE was performed to select the optimal variables, the number of selected variables was 301. That means the reduction rate of the variable number reached 85.49% (from 2074 to 301). Based on the variables selected by UVE, it was found that the UVE-LS-SVM model had a better performance in accuracy and robustness than the corresponding F-LS-SVM model (the LS-SVM model established based on full variables). The RMSEP value decreased by 23.6% from 2.264 to 1.729 after the UVE selection. Nevertheless, the informative variables selected by UVE were still too much. Therefore, SPA was further calculated based on the UVE selected variables to reduce redundancy of the informative variables. The results in Table 1 show that only 9 variables remained after the UVE-SPA variable selection. Moreover, the accuracy of the UVE-SPA-LS-SVM model was similar to the UVE-LS-SVM model, showing that the redundancy of the informative variables was successfully removed by SPA. At last, CARS was used to select the optimal variables from NIR data. After the variable selection of CARS, there were 26 variables left, which accounted for only 1.25% of the 2074 full variables. The established CARS-PLSR and CARS-LS-SVM models had similar results with the F-PLSR and F-LS-SVM models, as their RMSEP was 2.933, 2.264, 2.955, and 2.289, respectively. By comprehensively comparing the number of input variables, prediction accuracy, and robustness of different models, the best NIR model for predicting the storage days was the LS-SVM model based on the 9 optimal variables selected by UVE-SPA.

### 3.4. Prediction of Storage Days Based on MIR Spectra

#### 3.4.1. Calibration with Full MIR Variables

Similar to the NIR analysis, PLSR and LS-SVM models were first built based on full MIR variables, and the accuracy and robustness of these two models were compared. As shown in Table 2, both PLSR and LS-SVM models obtained good results with the *R*_P_^2^ values of 0.962 and 0.985, respectively. On the contrary, the robustness of these two models reached a satisfied level with the ABS values of 0.371 and 1.156 for the PLSR and LS-SVM models, respectively. It was noted that the results based full MIR variables were much accurate than that based on full NIR variables. The RMSEP of the PLSR model based on MIR data was only 63.96% of that based on NIR data, whereas the RMSEP of the LS-SVM model based on MIR data was only 52.25% of that based on NIR data. In terms of model robustness, the AB_RMSE values of both PLSR models based on MIR data and NIR data were less than 0.5, whereas the AB_RMSE of the LS-SVM model based on MIR data was only 51.08% of that based on NIR data. In general, the LS-SVM model was identified better than the PLSR model, when the full MIR variables were considered.

#### 3.4.2. Calibration with Selected MIR Variables

There were 1762 variables for the MIR data. To identify the important and informative variables from the MIR spectral region, the four variable selection strategies of SPA, UVE, UVE-SPA, and CARS were also implemented. It can be seen from Table 2 that there were 7, 230, 12, and 32 variables selected from the MIR spectral data by SPA, UVE, UVE-SPA, and CARS, respectively. Based on these selected variables, the PLSR and LS-SVM models were established and their results are shown in Table 2. In general, the accuracy of the LS-SVM models was better than that of the corresponding PLSR models. The average RMSEP value of the LS-SVM models was only 71.67% of that of the PLSR models (1.471 vs. 2.053). On the contrary, the average AB_RMSE values of the PLSR models and the LS-SVM models were similar (0.565 vs. 0.648). It shows that LS-SVM was much suitable than PLSR for the calibration of MIR data. Different from other three variable selection strategies, the result of CARS was not good. The RMSEP values of both CARS-PLSR and CARS-LS-SVM models were higher than corresponding models based on full MIR variables. The best model was the SPA-LS-SVM prediction model, which had *R*_P_^2^ and AB_RMSE values of 0.992 and 0.505, respectively. Compared with the F-LS-SVM model, the RMSEP and AB_RMSE values of the SPA-LS-SVM model decreased by 26.97% and 56.31%, respectively. Therefore, after the variable selection of SPA, both the accuracy and robustness of the LS-SVM model were improved. Moreover, only 0.39% of the full variables were selected by SPA (7 vs. 1762), showing that such improvement was achieved based on the most important variables. The successful variable selection could much reduce the computational complexity.

### 3.5. Prediction of Storage Days Based on the Combination of NIR and MIR Spectra

#### 3.5.1. Calibration with Full Variables of the Combination of NIR and MIR Spectra

To further explore the feasibility of NIR and MIR spectroscopic technique for the rapid identification of strawberry powder made from freshly available stored fruits, the NIR and MIR data were combined to generate a new spectral matrix to evaluate whether the identification capability could be improved. The related results are summarized and shown in Table 3. After spectral combination, there were a total of 3836 variables. The LS-SVM model based on the combined spectra with full variables had similar prediction result compared with that based on full MIR variables. On the contrary, the PLSR model based on the combined spectra with full variables had worse prediction than that based on full MIR variables. Therefore, the combination between NIR and MIR spectra did not improve the prediction.

#### 3.5.2. Calibration with Selected Variables of the Combination of NIR and MIR Spectra

Variable selection was further carried out to select optimal variables from the combined spectra and to evaluate if the prediction could be improved. As shown in Table 3, after the implementation of four variables selection strategies, there were 6, 731, 12, and 44 optimal variables selected from 3836 variables by SPA, UVE, UVE-SPA, and CARS, respectively, which accounted for 0.15%, 19.05%, 0.30%, and 1.15% of the full variables. Then, the PLSR and LS-SVM regression models were established based on the optimal variables, and the accuracy and robustness of prediction model were compared with full-variable models. It was found that the accuracy and robustness of the established models after screening variables were improved, compared with the full-variable model except the models established based on variables selected by CARS. Among these prediction models, the SPA-LS-SVM model was the best model for the prediction of storage days. Its *R*_P_^2^ and ABS values reached 0.989 and 0.522, respectively. Although the SPA-LS-SVM model had significant improvement in accuracy and robustness compared with the LS-SVM model based on full variables, it was not the best of all models. The accuracy and robustness of the SPA-LS-SVM model based on combined spectra was lower than that of the SPA-LS-SVM model based on MIR spectra (RMSEP values were 0.864 vs. 1.027 and AB_RMSE values were 0.505 vs. 0.522). Therefore, the combination of NIR and MIR data could not improve the accuracy and robustness of the prediction.

## 4. Discussion

By analyzing the RGB images of strawberry powder made from freshly available or the fruits stored at different days, it was difficult to identify them using naked eyes. NIR and MIR spectroscopy techniques were further used to distinguish whether the strawberry powder was made from the freshly available or the stored fruit. Good classification results of 100.00% correct rate were obtained. Therefore, although it was difficult to tell the freshly available and stored strawberry by visual observation on the powder, both MIR and NIR spectroscopic techniques accurately identified whether the strawberry powder was made from the freshly available or the stored fruit. Furthermore, to predict the storage days of strawberries that produce strawberry powder, the PLSR and LS-SVM models were established based on NIR, MIR, and NIR-MIR data with full variables or optimal variables, respectively. As shown in Tables 1–3, the best model was the SPA-LS-SVM model based on MIR data, which had the RPD value of 11.198, AB_RMSE value of only 0.505, and only 7 input variables. Actually, in general, the application of MIR spectroscopy data and NIR spectroscopy data for the prediction of strawberry powder storage days all achieved good results. When LS-SVM was used for the model calibration, all NIR and MIR models had RPD values higher than 4.0. The above results confirmed the feasibility of using NIR and MIR spectroscopic techniques for rapid identification of strawberry powder made from freshly available and stored fruits.

Two infrared spectral techniques were used in this work, namely, NIR spectroscopy and MIR spectroscopy. NIR and MIR spectra locate in different wavelength ranges, and their spectral data contain different information. Both of them are commonly used to determine the quality of agricultural products. To evaluate the predictive capabilities of two techniques used in this study, PLSR and LS-SVM models were established based on full spectral variables and selected optimal variables. In general, the performance of the models established based on the MIR and NIR data was different in terms of accuracy and robustness. When the models were established based on the NIR data, the RPD values were all above 2.8 and the RPD values of UVE-LS-SVM and UVE-SPA-LS-SVM models even reached 5.680 and 5.699, respectively. These results showed that the application of NIR spectroscopy in predicting the storage days of strawberry fruit to produce strawberry powder was feasible. When the MIR was considered to establish the models, better excellent performance was obtained than NIR. The average RPD values of the LS-SVM models were 4.825 and 7.577 for NIR and MIR spectra, respectively. The RPD values of the best models based on the NIR data set and MIR data set were 5.699 and 11.198, respectively. Therefore, MIR spectral data is more suitable for predicting strawberry powder storage days than NIR data. Moreover, although the spectral acquisition of NIR data was nondestructive, a certain amount of strawberry powder was needed to be taken out for the reflectance spectral collection, whereas the MIR spectral measurement with the ATR accessory only required only a small number of powder samples to be detected. Therefore, MIR spectroscopy was more suitable for predicting the storage days of the strawberries that the strawberry powder is made from. Nevertheless, the MIR instrument is too large to be portable. Therefore, when it is necessary to bring the testing equipment outdoors, NIR technique can be adopted, especially the predictive results of NIR spectra were also good (RPD value of the best model was higher than 5.0). In addition, MIR instruments are generally much more expensive than NIR instruments; therefore, the cost of the instruments is another main factor in selecting the two techniques.

To make full use of the spectral information of NIR and MIR techniques, this study not only compared the performance of the two infrared spectroscopic techniques, but also combined them together to build the PLSR and LS-SVM models. The results show that combination of the NIR data and MIR data did not improve the accuracy of the PLSR and LS-SVM models. Although the models established based on the combined spectra had better prediction than the corresponding models established based on the NIR spectra, there was no improvement after the combination, compared with the models established based on the MIR spectra. Therefore, simple combination of NIR and MIR data is not suggested in this work. Moreover, compared with the NIR spectra, the MIR spectra were the main contribution to the models based on the combined spectra, which indirectly verified the conclusion that MIR was more useful in the prediction of storage days.

Multivariable calibration is a key step in infrared spectrum analysis. To establish quantitative relationships between the spectral data and the storage days, two kinds of multivariate models (PLSR and LS-SVM) were established in this work, and their results were compared. When NIR spectra were considered, the average RPD value of all five PLSR models was 3.029, whereas that of all five LS-SVM models was 4.825. When MIR spectra was considered, the average RPD value of all five PLSR models was 5.005, whereas that of all five LS-SVM models was 7.577. When the combination of NIR and MIR spectra was considered, the average RPD value of all five PLSR models was 4.376, whereas that of all five LS-SVM models was 8.268. Moreover, the best models for three types of spectra (NIR, MIR, and the combination of NIR and MIR) were all established using LS-SVM. The above analysis indicates that the nonlinear calibration method of LS-SVM was much suitable than PLSR in predicting the storage days of the strawberries that the strawberry powder was made from.

The NIR and MIR spectral data had thousands of variables, and most of them were redundant and collinear. These useless variables not only increased the computational complexity of the model calibration, but also might reduce the accuracy and robustness of the models. To minimize the impact of these problems, four variable selection strategies of SPA, UVE, UVE-SPA, and CARS were adopted. In general, the results of models established based on the variables selected by CARS were worse than those of other methods. UVE obtained good results, but its selected variables were usually hundreds. In contrast, SPA and UVE-SPA selected only a few or a dozen variables, and the models they built could still maintain good precision and robustness. In addition, especially for LS-SVM models, variable selection could improve the robustness of the models for the most part. Therefore, it is recommended to apply the suitable variables selection strategy for spectral analysis.

As a preliminary study, the results show that it is feasible to identify strawberry powder made from freshly available and stored fruits by using the NIR and MIR spectroscopic techniques. However, further improvement should be made before the actual application. First, more strawberries from different varieties should be considered in future works. Second, only one storage condition of 0°C was studied in this work. In the future, different storage conditions should be considered. Nevertheless, the results of this study indicated that NIR and MIR techniques could identify the strawberry powder made from the fleshly available and the stored fruit, which was difficult to be identified by naked eyes. The outputs of this work are helpful to reduce fraud by using the long-term stored strawberries to make strawberry powder, which can provide consumers with quality-assured products to protect their legitimate rights and interests.

## 5. Conclusions

The feasibility of rapidly identifying whether the strawberry powder was made from freshly available or stored fruits and predicting the storage days was investigated using NIR and MIR techniques in tandem with chemometrics. The obtained results in this study show that both MIR and NIR could identify whether the strawberry powder was made from the freshly available or stored fruit with good classification results of 100.00% correct rate. Moreover, both MIR and NIR could predict the storage days of the strawberries that the strawberry powder is made from, and the MIR spectroscopic technique obtained better results than NIR. The SPA-LS-SVM model based on MIR spectra was the best model, which had the RPD value of 11.198 and AB_RMSE value of 0.505. In addition, LS-SVM was found to be more suitable than NIR for analyzing both NIR and MIR data. As the first investigation into the rapid identification of strawberry powder made from freshly available and stored fruits, the results in this study are very promising and should promote more effort to identify the raw material of fruit powder.

## Figures and Tables

**Table 1:** Prediction result of strawberry storage days based on NIR^n^ spectra with full variables and optimal variables.

Model	Variable selection	Variable number	Calibration			Prediction			RPD^l^	AB_RMSE^m^
			*r*_C^f^_	*R*_C^g^_^2^	RMSEC^h^	*r*_P^i^_	*R*_P^j^_^2^	RMSEP^k^		
PLSR^a^	Full-variable	2074	0.959	0.920	2.790	0.953	0.908	2.933	3.302	0.142
LS_SVM^b^	Full-variable	2074	1.000	1.000	0.001	0.973	0.945	2.264	4.302	2.263
PLSR	SPA^c^	3	0.958	0.918	2.816	0.937	0.877	3.386	2.855	0.570
LS_SVM	SPA	3	0.977	0.954	2.113	0.970	0.940	2.359	4.123	0.246
PLSR	UVE^d^	301	0.991	0.981	1.348	0.936	0.875	3.409	2.838	2.061
LS_SVM	UVE	301	0.996	0.991	0.913	0.984	0.968	1.729	5.680	0.816
PLSR	UVE_SPA	9	0.991	0.981	1.348	0.936	0.875	3.409	2.838	2.061
LS_SVM	UVE_SPA	9	0.996	0.992	0.872	0.985	0.968	1.723	5.699	0.852
PLSR	CARS^e^	26	0.978	0.956	2.073	0.953	0.906	2.955	3.314	0.882
LS_SVM	CARS	26	0.993	0.986	1.176	0.973	0.944	2.289	4.322	1.113

^a^Partial least squares regression, ^b^least squares support vector machines, ^c^successive projections algorithm, ^d^uninformative variable elimination, ^e^competitive adaptive reweighted sampling, ^f^correlation coefficients of calibration, ^g^determination coefficients of calibration, ^h^root-mean-square error of calibration, ^i^correlation coefficients of prediction, ^j^determination coefficients of prediction, ^k^root-mean-square error of prediction, ^l^residual prediction deviation, ^m^absolute difference between RMSEC and RMSEP, and ^n^near-infrared.

**Table 2:** Prediction result of strawberry storage days based on MIR^n^ spectra with full variables and optimal variables.

Model	Variable selection	Variable number	Calibration			Prediction			RPD^l^	AB_RMSE^m^
			*r*_C^f^_	*R*_C^g^_^2^	RMSEC^h^	*r*_P^i^_	*R*_P^j^_^2^	RMSEP^k^		
PLSR^a^	Full-variable	1762	0.988	0.977	1.505	0.981	0.962	1.876	5.155	0.371
LS_SVM^b^	Full-variable	1762	1.000	1.000	0.027	0.994	0.985	1.183	8.436	1.156
PLSR	SPA^c^	7	0.984	0.969	1.731	0.982	0.964	1.823	5.296	0.092
LS_SVM	SPA	7	0.999	0.999	0.359	0.996	0.992	0.864	11.198	0.505
PLSR	UVE^d^	230	0.994	0.988	1.094	0.984	0.968	1.718	5.621	0.625
LS_SVM	UVE	230	0.996	0.993	0.833	0.989	0.978	1.417	6.817	0.583
PLSR	UVE_SPA	12	0.994	0.988	1.094	0.984	0.968	1.718	5.621	0.625
LS_SVM	UVE_SPA	12	0.997	0.993	0.812	0.989	0.979	1.413	6.835	0.601
PLSR	CARS^e^	32	0.979	0.957	2.034	0.954	0.907	2.951	3.330	0.917
LS_SVM	CARS	32	0.991	0.983	1.288	0.976	0.948	2.190	4.600	0.902

^a^Partial least squares regression, ^b^least squares support vector machines, ^c^successive projections algorithm, ^d^uninformative variable elimination, ^e^competitive adaptive reweighted sampling, ^f^correlation coefficients of calibration, ^g^determination coefficients of calibration, ^h^root-mean-square error of calibration, ^i^correlation coefficients of prediction, ^j^determination coefficients of prediction, ^k^root-mean-square error of prediction, ^l^residual prediction deviation, ^m^absolute difference between RMSEC and RMSEP, and ^n^mid-infrared.

**Table 3:** Prediction result of strawberry storage day based on the combination of NIR^n^ and MIR^o^ spectra with full variables and optimal variables.

Model	Variable selection	Variable number	Calibration			Prediction			RPD^l^	AB_RMSE^m^
			*r*_C^f^_	*R*_C^g^_^2^	RMSEC^h^	*r*_P^i^_	*R*_P^j^_^2^	RMSEP^k^		
PLSR^a^	Full-variable	3836	0.990	0.980	1.404	0.967	0.934	2.473	3.942	1.069
LS_SVM^b^	Full-variable	3836	1.000	1.000	0.002	0.993	0.986	1.152	8.404	1.150
PLSR	SPA^c^	6	0.981	0.962	1.930	0.978	0.957	2.007	4.828	0.077
LS_SVM	SPA	6	0.999	0.997	0.505	0.994	0.989	1.027	9.397	0.522
PLSR	UVE^d^	731	0.996	0.991	0.920	0.983	0.965	1.818	5.334	0.899
LS_SVM	UVE	731	0.999	0.998	0.469	0.994	0.988	1.051	9.223	0.582
PLSR	UVE_SPA	12	0.994	0.987	1.109	0.976	0.950	2.168	4.525	1.058
LS_SVM	UVE_SPA	12	0.999	0.998	0.462	0.994	0.988	1.051	9.225	0.589
PLSR	CARS^e^	44	0.996	0.992	0.860	0.952	0.905	2.982	3.253	2.122
LS_SVM	CARS	44	0.998	0.996	0.603	0.981	0.961	1.906	5.089	1.302

^a^Partial least squares regression, ^b^least squares support vector machines, ^c^successive projections algorithm, ^d^uninformative variable elimination, ^e^competitive adaptive reweighted sampling, ^f^correlation coefficients of calibration, ^g^determination coefficients of calibration, ^h^root-mean-square error of calibration, ^i^correlation coefficients of prediction, ^j^determination coefficients of prediction, ^k^root-mean-square error of prediction, ^l^residual prediction deviation, ^m^absolute difference between RMSEC and RMSEP, ^n^near-infrared, and ^o^mid-infrared.

## Data Availability

The NIR and MIR data used to support the findings of this study have not been made available because this is a government research project that should not be made public.

## Nomenclature

NIR: Near-infrared
MIR: Mid-infrared
PLSR: Partial least squares regression
LS-SVM: Least squares support vector machines
SAP: Successive projections algorithm
UVE: Uninformative variable elimination
CARS: Competitive adaptive reweighted sampling
PLS-DA: Partial least squares discriminant analysis
*r*_C_: Correlation coefficients of calibration
*r*_P_: Correlation coefficients of prediction
*R*_C_^2^: Determination coefficients of calibration
*R*_P_^2^: Determination coefficients of prediction
RMSECV: Root-mean-square error of calibration
RMSEP: Root-mean-square error of prediction
RPD: Residual prediction deviation
AB_RMSE: Absolute difference between RMSEC and RMSEP.
